# Influence of exercise test on platelet function in patients with coronary arterial disease: A systematic review

**DOI:** 10.1097/MD.0000000000024932

**Published:** 2021-02-26

**Authors:** Chunhua Mo, Yanhui Wang, Zong Yue, Dayi Hu, Chun Yin

**Affiliations:** a Department of Cardiology, First Affiliated Hospital of Chongqing Medical University, Chongqing; b Cardiac Rehabilitation Center, Beijing First Hospital of Integrated Chinese and Western Medicine, Beijing; c Department of Cardiology, Chongqing General Hospital, University of Chinese Academy of Sciences, Chongqing, China.

**Keywords:** cardiac rehabilitation, Coronary artery diseases, exercise test, platelet function

## Abstract

**Background:**

Exercise test (ET) may have adverse effects on platelet function and induce acute thrombotic events in patients with coronary artery disease (CAD). The aim of this study is to investigate the platelet function and evaluate the risk of thrombotic events in CAD patients during ET.

**Methods:**

Pubmed, Embase, Cochrane Library, and Web of Science were searched for a systematic review from initiation to October 2019. The inclusion criteria were controlled clinical trails as study design; investigating platelet function in CAD patients during ET; with ET carried out by treadmill or bicycle ergometer; written in English. Included articles were screened based on title/abstract and full-text review by 2 independent reviewers. Platelet aggregation (PA), platelet surface expression of CD62p and PAC-1, plasma levels of platelet factor 4 (PF4) and beta-thromboglobulin (*β*-TG) were evaluated before and after ET.

**Results:**

Eighteen articles were included out of the 427 references initially identified. In most of the studies included ET was terminated because of limited symptoms. Prior to ET, no difference in platelet aggregation was observed in CAD patients compared with healthy controls in majority of the studies, with or without the treatment with Aspirin. Dual anti-platelet therapy suppressed adenosine diphosphate (ADP)-induced platelet aggregation at rest. After ET, platelet aggregation, the serum levels of β-thromboglobulin were found unchanged in majority of studies and platelet factor-4 were found unchanged in half of studies. The expression of platelet surface markers were elevated by ET in a few study.

**Conclusion:**

Symptom-limited exercise test did not affect platelet function in patients with coronary artery disease; however exercise to higher intensity may induce platelet activation.

## 1 Introduction

Comprehensive cardiac rehabilitation (CR) is associated with decreased mortality/morbidity and improved quality of life in patients with coronary artery diseases (CAD).^[1,2]^ Long-term exercise training as one of the core components of CR, plays essential roles on reducing the mortality and morbidity.^[3]^ However, acute exercise may lead to increased risk of myocardial infarction or cardiac arrest.^[4,5]^ CAD is a common type of cardiovascular disease, accounting for most exercise-related deaths in people aged over 40, in whom acute coronary artery plaque disruption and thrombotic occlusion were commonly found.^[37]^ Exercise test (ET) is commonly performed in CAD patients prior to the participation in a CR program to evaluate the exercise capacity of patients and develop an exercise prescription.^[9]^ This process could raise the concern that acute and sub-maximal/maximal stimulation during ET may increase thrombotic risk in CAD patients.

Platelets play crucial roles on the pathogenesis of atherosclerotic diseases during the formation of acute thrombus.^[38]^ Acute exercise might lead to platelet activation in healthy people and CAD patients, and activation of platelet could be involved in exercise-related thrombus formation.^[6]^ A few reviews have discussed platelet function during exercise, but with no confirm conclusion.^[6,7]^ The reasons of conflicting results were believed lies in lack of standardization across laboratories^[6,7]^ and spontaneous activation during analyzing procedures.^[33]^ In addition, former reviews have not distinguished studies investigating healthy individuals from studies of CAD patients, which might also contribute to the inconsistent results across studies. There was evidence that the risk of exercise-induced cardiac event was relatively higher in patients with CAD than healthy people.^[8]^ The effects of ET on platelet function in patients with CAD were not completely elucidated in previous studies, especially when the individuals were treated with antiplatelet drugs. Several methods are available to test platelet function, of which light transmission aggregometry is considered as the historical gold standard.^[39]^ Flow cytometry for the evaluation of platelet surface markers is an accurate method to analyze the activities in individual cells, and was increasingly used to investigate the effects of antiplatelet drugs.^[27]^ Limited information was available on the expression profiles of platelet surface markers in CAD patients following acute exercise.

In this systematic review, we summarized the evidence of the effects of ET on platelet function in CAD patients compared with controls. The aims of this review are as followed:

1. To investigate whether platelet function was impaired in CAD patients as compared with healthy controls before ET;
2. To evaluate postET function changes of platelet in patients compared with controls;
3. To examine the influences of anti-platelet drugs on exercise-induced platelet activation and
4. To identify possible factors associated with platelet function during or shortly after ET.

## 2 Methods

A systematic literature search was performed using Pubmed, Embase, Cochrane Library, and Web of Science (dated to 15/10/2019). The following search keywords were used: “exercise,” “exercise test,” “blood platelet,” “platelets,” “platelet activation,” “platelet function test,” “thrombocyte,” “thrombocyte function,” “thrombocyte activation,” “coronary artery disease,” and “controlled clinical trial”. A review protocol was created before the start of this study and the results are reported in accordance with the PRISMA guidelines.^[10]^ No patients or public were involved in this study. Ethics and patient consent are not applicable.

### 2.1 Study selection

Inclusion criteria were:

1. The studies including patients with coronary artery disease;
2. The studies investigating the influences of exercise test on platelet function;
3. Original researches;
4. Controlled clinical trials and prospective design.

Exclusion criteria were:

1. The studies investigating long-term exercise on platelet function;
2. The studies of exercise test carried out other than treadmill or bicycle ergometer;
3. Reviews,
4. Animal studies;
5. Guidelines;
6. Letters/comments without original data;
7. Book chapters;
8. Conference abstracts;
9. Protocols;
10. Case reports and
11. Non English articles.

### 2.2 Data extraction

After exclusion of duplicates, titles and abstracts of all identified studies were reviewed by 2 reviewers (Y.W. and C.M.) using data extraction forms. Full-text articles were screened by the same 2 reviewers. Discrepancies were resolved by consulting a third reviewer (C.Y.). The risk of bias was assessed for each randomized controlled trial (RCT), according to the Cochrance Collaboration's tool.^[18]^

### 2.3 Outcome measures

Platelet function was compared before and shortly after ET:

1. Platelet aggregation (PA) tested by light transmission aggregometry;
2. Platelet surface markers of platelet activation: P-Selectin (CD62p) and activated complexes of integrin *α*IIb*β*3 (procaspase activating compound 1, PAC-1);
3. Soluble platelet activation markers: plasma levels of platelet factor 4 (PF4) and beta-thromboglobulin (*β*-TG).

## 3 Results

### 3.1 Study and patient characteristics

**Figure 1:**
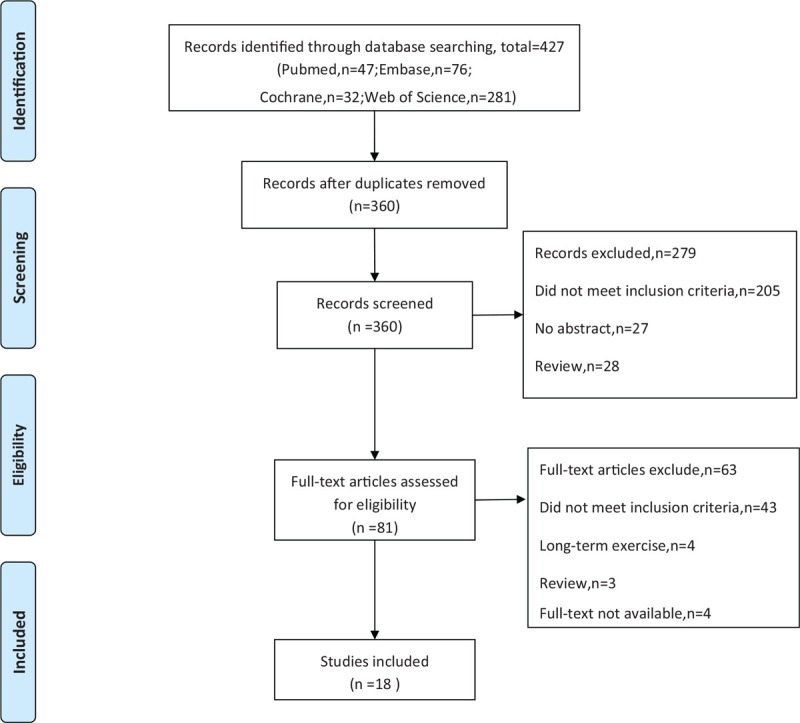
Flow chart of the study inclusion and exclusion process.

The processes of study screening were illustrated in Figure 1. A total of 427 studies were identified by electronic searches, 67 of which were duplicates. After reading the title and abstract of the identified studies, 279 studies were discarded for not fulfilling the inclusion criteria. The resulting 81 articles were screened by reading full-text, 63 of which were excluded for not fulfilling the inclusion criteria. Finally, 18 articles^[11–17,19–26,28–30]^ were included in this review. Sixteen studies were not randomized designed, 2 RCTs^[11,15]^ were assessed for the risk of bias. Low risk in selection bias and unclear risk in reporting bias were found. A meta-analysis was not able to be conducted.

To investigate platelet function, majority of these studies examined PA with light transmission aggregometry.^[11–17]^ Soluble platelet release markers: *β*-TG,^[12,13,19–23]^ PF4^[13,19–21,23,24]^ were also wildly used in researches. Some studies investigatied the expression of P-selectin (CD62p),^[15,25,26]^ GPIIb/IIIa (CD41)^[26]^ or PAC-1^[25]^ on platelet membrane with flow cytometry, which provide important information on individual cells.^[27]^ PFA-100 was also used in few studies.^[25,28]^

Among the studies, 8 of them used a bicycle ergometer test,^[11,13,15,16,20,21,26,29]^ starting at a load of 25w-50w and increased by 20w-25w/2minuts; and ten studies used treadmill exercise according to standard or modified Bruce protocol.^[12,14,17,19,22–25,28,30]^ Limited symptom or heart rate >85% predicted or ECG change or exhaust was used in most studies as terminate criteria in patients group, while exhaust was used in healthy controls. Some studies were performed during drug treatments, whereas the others were carried out after the discontinuation of drugs.^[20,21,30]^

### 3.2 The effects of ET on platelet aggregation

Prior to ET, most studies revealed that platelet aggregation was not different between CAD patients and healthy controls (Table 1), with or without the treatment with Aspirin.^[12,13,17,25,28]^ One study indicated strengthened platelet aggregation only in patient group.^[14]^ After ET, platelet aggregation remained unchanged in 3 studies,^[12,17,28]^ and was increased in 2 studies,^[13,14]^ and decreased in 1 study^[25]^ within patients, and was unchanged in 4 studies^[12,17,25,28]^ and increased in 2 studies^[13,14]^ within healthy controls. In addition, platelet aggregation was not different between patients and controls in most studies after ET.^[12,13,17,25,28]^ The study that revealed enhanced platelet aggregation in patients prior to ET also suggested elevated platelet aggregation following ET.^[14]^

**Table 1:** Studies on the effects of ET on platelet aggregation in patients with CAD and controls (n = 9).

	Study population		Anti-platelet drugs		Indications for termination of ET	Platelet aggregation			
Author, date	Group 1, n	Group 2, n	Group 1	Group 2		Group 1 vs 2		After vs Before ET	
						Before ET	After ET	Group 1	Group 2
Mehta et al, 1982^[12]^	CAD, 22	Healthy, 13	**-**	**-**	Severe angina; ST-segment depression >3 mm;	No	No	No	No
Yoshida et al, 1983^[17]^	CAD, 20	Healthy, 15	**-**	**-**	Severe angina; Significant ST depression; Exhaustion; HR reached at least 85% of the maximal predicted.	No	No	No	No
Wallen et al, 1997^[13]^	CAD, 113	Healthy, 50	ASA	**-**	Symptom-limited; Exhaustion.	No	No	Higher	Higher
Aurigemma et al, 2007^[25]^	CAD, 26	Healthy, 10	ASA	**-**	Angina; ST-segment depression >3 mm; Exhaustion; Clinically relevant events.	No	No	Lower	No
Pamukcu et al, 2005^[28]^	CAD, 62	Healthy, 20	ASA	ASA	Symptom limited.	No	No	No	No
Kuliczkowski et al, 2007^[14]^	CAD, 40	Healthy,10	ASA	ASA	Angina; ST-segment depression >2 mm; Exhaustion; HR of sub-maximal exercise reached.	Higher	Higher	Higher	Higher
Kitai et al, 2001^[16]^	CAD, 16	CAD, 16	ASA	**-**	Angina, ST-segment depression >2 mm, Exhaustion; HR obtaining at least 85% of the maximal predicted.	No	No	Higher	Higher
Kitai et al, 2001^[16]^	CAD, 16	CAD, 16	ASA+Ticlopidine	**-**		Lower	Lower	No	Higher
Perneby et al, 2007^[15]^	CAD, 16	CAD, 15	ASA+Clopidogrel	ASA	Severe angina, ST-segment depression>4mm; Exhaustion.	Lower	Lower	No	No
Brunner et al, 2018^[11]^	CAD,10	CAD, 10	ASA+Clopidogrel	ASA	Moderate-to-severe angina; ST-segment elevation >1 mm or depression >2 mm; Exhaustion.	Lower	Lower	Higher	Higher
Brunner et al, 2018^[11]^	CAD, 11	CAD,10	ASA+Prasugrel	ASA		Lower	Lower	Higher	Higher

Group 1, test group in each study; Group 2, control group in each study; CAD = coronary heart disease, ASA = Aspirin, No = no significant difference, ET = exercise testing; HR: heart rate; “-”: not used.

Kitai et al revealed no difference of platelet aggregation between CAD patients with and without Aspirin treatment prior to ET (Table 1), and enhanced platelet aggregation in both groups after ET.^[16]^ Platelet aggregation was significantly inhibited in CAD patients with dual anti-platelet treatment (DAPT, Aspirin in combination with Clopidogrel, Prasugrel or Ticlopidine) compared with mono anti-platelet treatment (Aspirin alone) before and after ET.^[11,15,16]^ In addition, to compare the postET platelet aggregation to baseline within each group, Brunner et al indicated an increased tendency both in DAPT and Aspirin alone group^[11]^; however, Perneby et al revealed no change in both groups,^[15]^ whereas Kitai et al detected enhanced aggregation in Aspirin alone group.^[16]^

### 3.3 The effects of ET on the levels of β-thromboglobulin and platelet factor-4

Some studies revealed increased *β*-TG level in patients before exercise by compared with healthy controls,^[12,20,22]^ while the others suggested no difference between experimental groups^[19,21,23]^ (Table 2). Following exercise, the level of *β*-TG was increased in 2 studies,^[12,13]^ and remained unchanged in 5 studies^[19–23]^ in CAD patients. The expression of *β*-TG was elevated in 4 studies^[12,13,19,20]^ and unchanged in 3 studies^[21–23]^ in healthy controls compared to baseline. The level of *β*-TG was decreased in patients with CAD in 2 studies,^[19,20]^ increased in 2 studies^[12,22]^ and unchanged in three studies^[13,21,23]^ following ET.

**Table 2:** Studies on the effects of ET on *β*-thromboglobulin and platelet factor-4 in CAD patients (n = 8).

	Study population			Group 1 vs. 2		After vs. Before ET	
Author, date	Group 1, n	Group 2, n	Soluble markers of platelet function	Before ET	After ET	Group 1	Group 2
Stratton et al, 1982^[19]^	CAD, 25	Healthy, 10	*β*-TG	No	Lower	No	Higher
Schernthaner et al, 1983^[20]^	CAD, 53	Healthy, 9	*β*-TG	Higher	Lower	No	Higher
Mehta et al, 1982^[12]^	CAD, 22	Healthy, 13	*β*-TG	Higher	Higher	Higher	Higher
Marcella et al, 1983^[21]^	CAD, 13	Healthy, 10	*β*-TG	No	No	No	No
Hughes et al, 1982^[22]^	CAD, 18	Healthy, 26	*β*-TG	Higher	Higher	No	No
Wallen et al, 1997^[13]^	CAD, 113	Healthy, 50	*β*-TG	No	No	Higher	Higher
Strauss et al, 1985^[23]^	CAD, 24	Healthy, 9	*β*-TG	No	No	No	No
Stratton et al, 1982^[19]^	CAD, 25	Healthy, 10	PF4	No	Lower	Higher	Higher
Schernthaner et al, 1983^[20]^	CAD, 53	Healthy, 9	PF4	Higher	Lower	No	Higher
Marcella et al, 1983^[21]^	CAD, 13	Healthy, 10	PF4	Higher	No	No	No
Levine *et al*, 1984^[24]^	CAD, 84	Healthy, 10	PF4	Higher	Higher	Higher	No
Wallen et al, 1997^[13]^	CAD, 113	Healthy, 50	PF4	No	No	Higher	Higher
Strauss et al, 1985^[23]^	CAD, 24	Healthy, 9	PF4	No	No	No	No

CAD = coronary heart disease, *β*-TG,*β*-thromboglobulin, PF4 = platelet factor-4, No = no significant difference, ET = exercise testing.

Prior to ET, 3 studies revealed increased level of PF-4 in patients compared with healthy controls^[20,21,24]^ and 3 studies indicated no difference between the 2 groups^[13,19,23]^ (Table 2). Compared with baseline after ET, elevated PF-4 was detected in 2 studies within both patient and control groups,^[13,19]^ in 1 study within patient group alone,^[24]^ and in 1 study within control group alone.^[20]^ The expression level of PF-4 was decreased in CAD patients compared to controls in 2 studies,^[19,20]^ increased in 1 study^[24]^ and unchanged in 3 studies following ET.^[13,21,23]^

### 3.4 The influences of ET on the expression of platelet surface markers

Aurigemma et al suggested that the mean fluorescence intensity (MFI) of PAC-1, CD41, and CD62p on the platelet surface were not different between CAD patients and healthy controls prior to ET, and increased only in CAD patients after ET^[25]^ (Table 3). Lindemann et al revealed reduced rates of CD41^+^ and CD62p^+^ platelets in CAD patient compared with controls before and after ET, while decreased rates of CD41^+^ and CD62p^+^ platelets were detected postET in CAD patients; however, no effects were observed in healthy controls.^[26]^ Perneby et al compared the influences of anti-platelet drugs on exercise-induce platelet CD62p in CAD patients, and the results suggested that DAPT but not Aspirin suppressed adenosine diphosphate (ADP)-induced CD62p^+^ platelet augmentation before and after ET, but both drug regimens failed to decrease the expression of exercise-induced platelet CD62p, which were in consistence with the findings within platelet aggregation.^[15]^ In addition, Sanguigni et al revealed that Amlodipine partially inhibited the exercise-induced augmentation of CD62p^+^ platelet in patients with CAD.^[29]^

**Table 3:** Previous reports on the effects of ET on the levels of platelet surface makers in patients with CAD (n = 4).

	Study population		Anti-platelet drug			Group 1 vs 2		After vs. Before ET	
Author, date	Group 1, n	Group 2, n	Group 1	Group 2	Surface markers of platelet function	Before ET	After ET	Group 1	Group 2
Aurigemma et al, 2007^[25]^	CAD, 26	Healthy, 10	ASA	**–**	CD62p(MFI)	No	not reported	Higher	No
Aurigemma et al, 2007^[25]^	CAD, 26	Healthy, 10	ASA	**–**	PAC-1(MFI)	No	not reported	Higher	No
Lindemann et al, 1999^[26]^	CAD, 12	Healthy, 11	ASA	ASA	CD62p/CD61	Lower	Lower	Lower	No
Perneby et al, 2007^[15]^	CAD, 16	CAD, 15	ASA+Clopidogrel	ASA	CD62p/CD42a	No	No	Higher	Higher
Sanguigni et al, 1999^[29]^	CAD, 10	CAD,10	Amlodipine	**–**	CD62p(% of +cell)	No	Lower	Higher	Higher

CAD = coronary heart disease, ASA = Aspirin, No = no significant difference, ET = exercise testing, PAC-1 = procaspase activating compound 1, MFI = mean fluorescence intensity, “-”: not used.

## 4 Discussion

In this systematic review, we focused on the platelet function changes in CAD patients during or shortly after ET, and tried to identify potential risk factors that may affect exercise-induced platelet activation, which are essential for clinical practice. Our study found out that following symptom-limited ET, platelet aggregation, the serum levels of β-TG were found unchanged in majority of studies and PF-4 were found unchanged in half of studies, which indicated that the platelet function of CAD patients were not affected by ET in most cases.

Our result was not totally consistent with a former review^[6]^ which concluded that acute and strenuous exercise can lead to platelet activation. The explication may lies in the differences of study population across researches. The former review^[6]^ included studies of healthy individuals and athletes, who performed exhaustive exercise and resulted in significant platelet activation. For example, one of the study reported significant increase in platelet aggregation and platelet surface markers expression by exercise test in football referees who had achieved mean peak VO_2_ of 47.33 ml/minutes kg.^[35]^ Most studies included in our review used symptom limitation as terminate criteria of ET. Patients with CAD was less likely to achieve a exercise capacity during ET as healthy people or athletes did, because of pathophysiologic reasons (e.g., myocardial ischemia during ET), psychological problems (e.g., depression) and the influence of drugs (e.g., beta-adrenegic receptor blockers) in CAD patients. It has been revealed that the intensity, duration and exercise capacity of acute exercise could affect the platelet response to exercise.^[31]^ Symptom-limited ET did not reached the intensity to result in platelet activation in CAD patients, and angina pectoris played a protective role of in this situation.^[36]^

The treatment with Aspirin exhibited no significant effects on platelet aggregation in CAD patients compared with patients without treatment or healthy controls; however, the combination of Aspirin with Clopidogrel or Prasugrel significantly reduced ADP-induced platelet aggregation in CAD patients before and after ET. The underlying mechanisms could be that Aspirin functions through irreversible inhibition of platelet cyclooxygenase-1 and can not affect ADP-induced platelet aggregation, whereas inhibitors of platelet ADP receptor P2Y12 that can significantly influence ADP-induced platelet aggregation.^[11]^ Aspirin alone or in combination with Clopidogrel or Prasugrel did not attenuate exercise-induced platelet aggregation in CAD patients; however, the combination of Aspirin with Ticlopidine significantly suppressed shear ADP-, collagen- and stress-induced aggregation before and after exercise, and this combination could work better in preventing thrombotic events during ET in CAD patients.^[16]^

Drugs used for secondary prevention of CAD may affect platelet function during ET. It has been reported that beta-blockers could reduce basal heart rate and heart response to exercise, which may decrease heart workload and increase exercise capacity.^[32]^ Amlodipine also exhibited a favorable effect on exercise-induced platelet activation within CAD patients.^[29]^ Additionally, underappreciated differences of drug regime across various study groups may contribute to the conflicting results, and more studies of drug effects (e.g., anti-hypertensive and anti-diabetic drugs, statins, nitrates, etc.) on exercise-induced platelet activation are required. Moreover, withdrawal of beta-blockers prior to ET may lead to heart rate rebound and platelet activation.^[32]^ The drugs were terminated before ET in some studies^[20,21,30]^ might lead to platelet hyper-reaction and increase the risk of thrombotic events. The stability and continuation of drug usage could contribute to well-controlled heart rate, blood pressure and blood glucose before ET and may be a potential method to reduce the thrombotic risk in CAD patients.

Augmented platelet count was considered as an independent risk factor of thrombotic events.^[34]^ ET may induce increased platelet count in both CAD patients and healthy controls,^[11–13,25]^ sympathetic stimulation triggered by ET could serve essential roles in this above-mentioned process.^[33]^ Exercise can transiently elevate the concentration of epinephrine, subsequently inducing the release of platelets from the liver, lungs and spleen and resulting in platelet count augmentation.^[40,41]^ These processes could rarely be affected by antiplatelet drugs, and this may explain why antiplatelet was not able to inhibit exercise-induced enhancement of platelet aggregation. Platelet density can significantly influence platelet aggregation.^[13]^ Efforts were made in some study^[16]^ to balance the platelet count in platelet rich plasma before aggregation, which could underestimate the post-exercise thrombotic risk.

This study provides a comprehensive review on the influence of exercise test on CAD patients. It provides detailed information on study population, evaluation of platelet function, and principle results on platelet function before and after exercise. However, there are still some limitations in the present study. This review indicated a relationship between exercise intensity and platelet function, however, no quantitative analyze was conducted, because the information of exercise intensity was not available in most of these abovementioned studies. There may exist a threshold of exercise intensity above which an ET would induce significant platelet activation, which should be taken into consideration for further investigations. Only the influences of ET were investigated in this review, thus the effects of long time exercise training in CR programs on platelet function were not evaluated, which need to be ruled out in future investigations. Few studies examined the influences of drugs on exercise-induced platelet activation, which fulfills the inclusion criteria, so it is not possible to determine the effects of drugs on exercise-induced platelet activation. A meta-analysis could be carried out in future work due to the heterogeneity of previous studies and the large number of anti-platelet drugs used across the studies.

## 5 Conclusion

Symptom-limited exercise test did not affect platelet function in patients with coronary artery disease, and was relatively safe to perform, however exercise test to higher intensity may induce platelet activation. Furthermore, in patients with coronary artery disease the effects of drugs on platelet function during exercise test should be taken into consideration for further investigations.

## Author contributions

**Conceptualization:** Chun Yin.

**Data curation:** Chunhua Mo, Yanhui Wang.

**Formal analysis:** Chunhua Mo, Yanhui Wang.

**Funding acquisition:** Chun Yin.

**Investigation:** Chunhua Mo, Yanhui Wang.

**Methodology:** Chun Yin.

**Project administration:** Dayi Hu.

**Resources:** Zong Yue.

**Software:** Yanhui Wang, Zong Yue.

**Supervision:** Dayi Hu.

**Validation:** Dayi Hu.

**Visualization:** Dayi Hu.

**Writing – original draft:** Chunhua Mo, Yanhui Wang.

**Writing – review & editing:** Chunhua Mo, Yanhui Wang, Chun Yin.
